# The Use of the Great Toe Pulp Free Flap in Dystrophic Fingertips

**DOI:** 10.3390/jpm15020044

**Published:** 2025-01-23

**Authors:** Alessandro Crosio, Mauro Magnani, Simona Odella, Matilde Cacianti, Francesco Maria Locatelli, Pierluigi Tos

**Affiliations:** Department of Hand Surgery and Reconstructive Microsurgery, Gaetano Pini-CTO Hospital, 20122 Milan, Italy; alessandro.crosio@unito.it (A.C.); mauro.magnani@asst-pini-cto.it (M.M.); simona.odella@asst-pini-cto.it (S.O.); matilde.cacianti@asst-pini-cto.it (M.C.); francescomaria.locatelli@asst-pini-cto.it (F.M.L.)

**Keywords:** great toe pulp, free flap, dystrophic finger, reconstructive surgery

## Abstract

**Background**: Lesions of the digital apices are common, and several treatment strategies can be considered for them. Among these, the free great toe pulp flap can be used. **Methods**: This is a retrospective report in which five patients undergoing hallux free flap surgery for loss of pulpal substance at the level of the hand were evaluated. They were re-evaluated by using both clinical testing to assess sensitivity and the use of questionnaires to estimate function. **Results**: None of the performed flaps failed. The mean follow-up was 36 months (range 16–66 months). With SW-MF, the mean value was 3.734 compared to 2.986 for the same contralateral finger. The S2-PD test attested a mean value of 6.8 mm (range 6–8 mm) in contrast to the contralateral finger, which showed a mean result of 3.2 mm (range 3–5 mm), while the D-2PD indicated lower values for both the operated finger, with a mean value of 6.4 mm (range 4–8 mm), and the healthy finger. **Conclusions**: When a dystrophic fingertip results from an inappropriate acute management, the GTP flap appears to be an excellent strategy to restore the specialized tissue of finger pulp and to bring supple tissue to the correct PIP flexion contracture or the small first web space contracture. It is mostly required for thumb and radial fingers’ reconstruction, especially in young patients or those who need high functional demands and/or present an extensive loss of substance that cannot be resolved with local flaps.

## 1. Introduction

Traumatic fingertip injuries are the most common injuries to the hand. Management strategies for these injuries depend on the site and degree of tissue loss in the wounds and vary by country or region [1,2,3,4,5,6,7,8,9]. Hand and finger injuries can be disabling and affect all ages. In adults, these injuries are commonly due to work activities. In this context, lacerations constitute the main type of injury, followed by crush and avulsion injuries. Different treatment options may be considered on the basis of the injury’s different features, such as extent, bone exposure, functional demands, and the comorbidities of the patient. Treatments vary from healing by second intention for less extensive injuries to local or free flaps for lesions with bone exposure. In a large number of cases, healing by secondary intention could end with valid restoration of the fingertip, but when the loss is wide and deep, a dystrophic scar can be expected. In such cases, according to the width of skin loss, local flaps can be planned. When a large amount of soft tissue has to be replaced, respecting the like-to-like principle [10], the use of a great toe pulp (GTP) flap, as described by Buncke and Rose [11] in 1979, is a widespread option. This procedure should restore the pulp contour and discriminative sensitivity [12] as much as possible, also taking into consideration the aesthetic result [10] and, therefore, the harvest sites.

In the current literature, this type of procedure is used in the acute setting for covering lesions with loss of digital pulp, but it is not clear in which cases it has to be used. In fact, the literature reports several papers in which this flap has been used as the first choice for lesions of different sizes, even with small extensions. Furthermore, use of the GTP has not been reported since then in the treatment of chronic pulp lesions of the digital apices. This report was conducted to describe the possibility of reconstructing a dystrophic fingertip with a microvascular GTP flap transfer, assessing the recovery of motion, sensation, and patient satisfaction.

## 2. Materials and Methods

A total of 5 patients were retrospectively evaluated and treated in the Department of Hand Surgery and Reconstructive Microsurgery of ASST G. Pini-CTO between January 2017 and June 2023, who underwent the surgical procedure of pulpal substance loss reconstruction due to chronic dystrophic sequelae after traumatic injuries. The surgical procedure involved the use of a GTP flap. In November 2023, patients were re-evaluated clinically through clinical tests and evaluation questionnaires in order to assess the recovery of sensation and satisfaction and statistically analyzed by determining the mean and median of the results. Ethical review and approval were waived for this study due to its retrospective nature (art. 110 d.lgs 196/2003 revised 2024 measure 298).

### 2.1. Surgical Procedure

Before surgery, a Doppler study was carried out to determine whether the main artery to the toe was dorsal or plantar.

The operation was performed under general anesthesia by two teams. One was dedicated to the preparation of the recipient site at which the chronic lesion was first removed, allowing for a recovery of the range of motion (ROM), and then the recipient vessels and nerve were prepared. The second team designed the GTP flap according to the defect in the toe. An incision was made in the first intermetatarsal space to identify the dorsal superficial veins of the hallux and was extended proximally for an appropriate length. The flap was harvested retrogradely by microsurgical dissection using 3.5× loupes, including the lateral neurovascular bundle as a pedicle, with the length of the pedicle determined by the location of the recipient vessels. The flap was transferred to cover the finger pulp defect. The recipient artery was one of the proper digital arteries according to their location and flow. In all cases the proper artery was good enough and it was not required to move further in the proximal direction to find a feasible artery. The vein was identified on the dorsum of the hand or the finger, and the vein of the flap was tunnelized from volar to dorsal until it reached the recipient one. Using the microscope, first the arterial anastomosis was performed, then the venous one. For the latter, when more than one venous branch was present, the choice was made after the tourniquet release on the basis of venous outflow. The anastomoses were performed end-to-end with an 8-0 or 9-0 nylon suture. Finally, neurorrhaphy between the sensory nerve of the great toe and the ulnar proper digital nerve of the thumb or the radial digital nerve of the index finger was performed with an 8-0 nylon thread. In most of the cases, the donor site was reduced as much as possible, and the remnant was left healing by secondary intention. Flap circulation was carefully monitored by color, temperature, and capillary refill. In the post-operative period, anticoagulation prophylaxis was administered with Enoxaparin sodium 4000 iu SQ 1 once a day. The patient rested in bed for at least 4–5 days after surgery; an hourly check of capillary refill and temperature was performed for the first 48 h, then 3 times a day until the 5th post-operative day.

### 2.2. Outcomes Assessment

The Semmes–Weinstein monofilaments and static discrimination test (Weber’s Test) (S-2PD) in the motion of the 2 points (D-2PD), according to Dellon [13], were compared with the contralateral side were used for sensitivity assessment.

The ROM of the metacarpophalangeal, proximal interphalangeal, and distal interphalangeal joints was evaluated.

The donor and harvest site complications were also recorded.

For pain assessment at the operated finger and donor site, the VAS scale (0–10) was used.

For functional assessment, the Quick-DASH questionnaire (q-DASH) and the Michigan Hand Outcomes questionnaire (MH-O) were used, the latter of which also allowed for assessment of patient satisfaction from an aesthetic point of view.

The Cold Intolerance Symptom Severity (CISS) questionnaire was used to assess cold intolerance symptoms of the operated finger in activities of daily living, occupational difficulties, and inability to perform recreational activities.

Through the Foot Functional Index assessment (FF-I), donor site symptomatology and interference in with daily living activities and work were measured.

Finally, patients’ overall satisfaction (O-S) with the outcomes of this surgery was assessed with a scale from 0 (not at all satisfied) to 10 (completely satisfied).

## 3. Results

None of the flaps performed failed, the last patient suffered an arterial thrombosis on post-operative day 3 and required anastomosis revision. Due to the enlargement of the flap secondary to arterial complication, the edges healed by secondary intention, but without any consequence for the overall result. The recipient vessels have been the digital ones for all cases, and in all patients a nerve anastomosis was performed.

Three patients presented a dystrophic fingertip as a result of conservative treatment with serial dressings. None of them were treated with local flap in acute cases. This induced not only a dystrophic scar, but also a flexion contracture of the fingertip that limited the ROM. In fact, following dystrophic scar resection, the gap was enlarged to solve flexion contracture or abduction limitation of the thumb, inducing quite a large loss of soft tissue. In this series, the flap required was 3 cm long and about 2 cm wide with a total surface of 6 cm^2^. One patient required an immediate Z-plasty, in the other one this was performed a few months later.

The operative time was around 300 min, and the procedure was performed by two teams. Usually, the patient stays in the hospital for 7 days due to bed rest and flap monitoring. When a major complication occurred (arterial thrombosis), the inpatient time increased to 25 days because of a new surgical procedure and monitoring time.

At outpatient evaluation, all flaps were trophic and well-perfused. The mean follow-up was 36 months, with a range of 12–66 months (Table 1).

The measurement of the joint ROM of the MPs indicated no difference between the fingers of the two hands, showing a full range of motion. At the level of the PIP and DIP, the ROM in flexion was about 10° less than the contralateral finger.

The sensitivity was evaluated by two tests. With SW-MF, the mean value was 3.734 compared with 2.986 for the same contralateral finger. The S2-PD test attested a mean value of 6.8 mm (range 6–8 mm) in contrast to the contralateral finger, which showed a mean result of 3.2 mm (range 3–4 mm), while the D-2PD showed lower values for both the operated finger with a mean value of 6.4 mm (range 4–8 mm) and the healthy finger.

**Figure 1:**
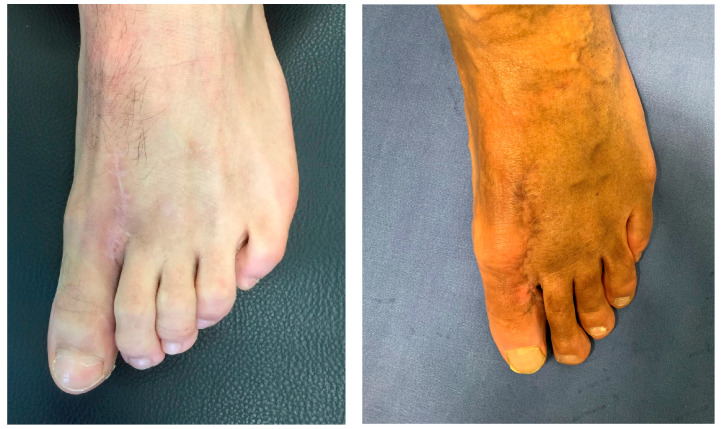
Two examples of donor sites at follow-up showing complete healing.

The donor site (Figure 1) with a VAS of 0, except in the winter seasons where the VAS at the level of the operated finger was 3, particularly on days with colder temperatures. Cold intolerance investigated through the CISS, in fact, shows a mean value of 13.6 (range 10–18).

The operated hand function assessed with the qDASH and the MH-O questionnaires showed no difficulties in the activities of daily living. Moreover, the MH-O revealed high patient satisfaction for aesthetic outcomes. Finally, the FF-I assessment reported no problems from patients.

The overall patient satisfaction with the result obtained was very high with a mean value of 9.2 (Table 2).

### 3.1. Case 1

**Figure 2:**
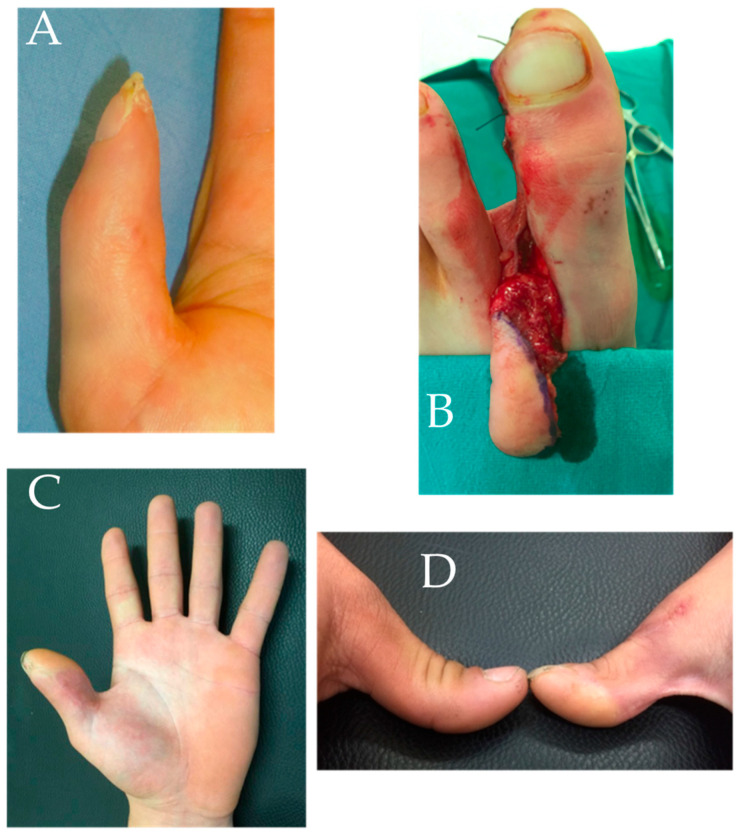
Case report 1: (**A**) preparatory image showing hypotrophy of the thumb pulp; (**B**) intra-operative picture of flap harvesting; (**C**) post-operative result; and (**D**) comparison with contralateral thumb.

A 20-year-old male patient presenting with atrophic pulpal substance at the level of the thumb of the right hand (dominant) with a flexion contracture of the interphalangeal joint (IPj) of the thumb. He was previously treated with serial occlusive dressings until complete re-epithelialization. Approximately 12 months after the injury, pulp reconstruction surgery with a GTP flap was performed; arterial anastomosis had to be executed three times during surgery due to repeated spasms of the artery that did not allow an adequate flow. Thereafter, no further revision surgery was necessary. The patient complained the most about hyperalgesia, which resolved completely after surgery (Figure 2).

### 3.2. Case 2

A 17-year-old female patient presented with a proximal interphalangeal joint (PIPj) flexion contracture associated with pulpal hypotrophy of the index finger of the right hand (non-dominant) in the aftermath of crush trauma following a fall from a horse. The patient complained of pain and the inability to extend the finger, and also presented with a retracting scar on the middle finger and the claw of the nail apparatus of the index finger. This was the consequence of a crush injury in which no local flaps were necessary and it was healed by serial dressings.

**Figure 3:**
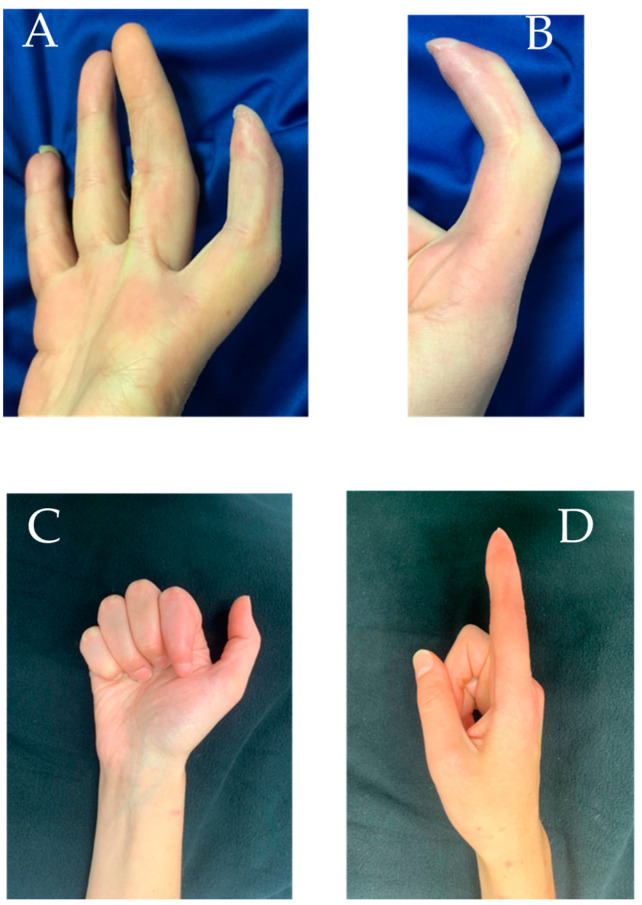
Case report 2: (**A**,**B**) pre-operative images showing hypotrophy of the index pulp with significant scar retraction; (**C**,**D**) post-operative images in which a good result in flexion and full recovery of index extension are assessed.

She underwent pulp reconstruction surgery with a GTP flap and Z-plasty to solve the flexion contracture of the PIPj 16 months after the injury (Figure 3).

### 3.3. Case 3

**Figure 4:**
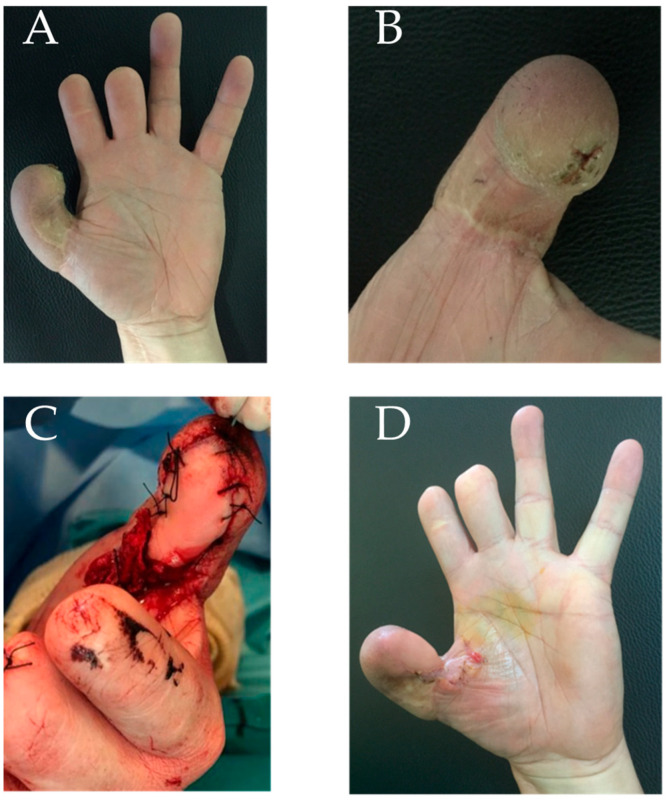
Case report 3: (**A**) pre-operative hand; (**B**) detail of chronic ulcer at thumb flap level; (**C**) intraoperative image at the end of the surgical procedure; and (**D**) final result at follow-up.

A 38-year-old male patient presented with a chronic ulcer at the level of the thumb of the right hand (dominant) in the outcomes of a reverse radial flap used to reconstruct the thumb about 12 years earlier following a crush trauma. A chronic ulcer was present on the volar surface of the flap, the area of maximal stimulation during thumb usage. No signs of infection were present. Moreover, a mild adduction contracture of the first web space was noted. The scarred area was removed, this permitted us to open the web space. The loss of substance was replaced with a GTP flap that covered the space between the thumb apex and base as shown in Figure 4. No further intervention was required. Sensitivity also improved significantly, providing a better hand function.

### 3.4. Case 4

**Figure 5:**
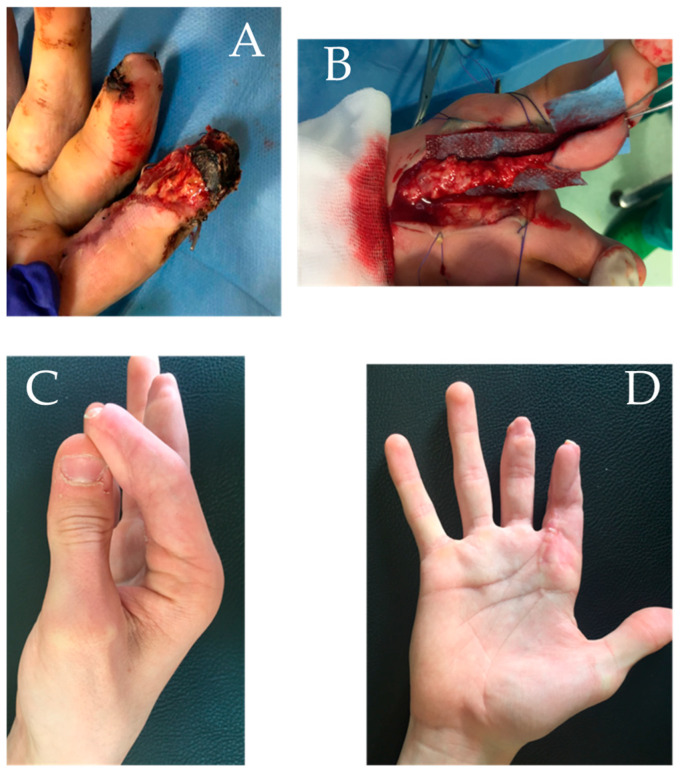
Case report 4: (**A**) pre-operative image following removal of necrotic tissue; (**B**) intra-operative image of flap harvesting; (**C**) post-operative thumb–index pincer grip; (**D**) final result at follow-up.

A 15-year-old male rally driver patient presented with traumatic sub-amputation of the index and middle fingers of his right hand (dominant) following a motor vehicle accident during a race. An urgent revascularization was attempted, but the fingertip became progressively necrotic with tendon exposure. Two months after the trauma, in order to not shorten the index finger considering the age and functional requirement of the patient, pulp reconstruction was performed. No local flaps were available for such a large loss, so a GTP flap was planned. A few months after reconstruction, a Z-plasty was required to solve a retracting scar that limited PIPj extension. The functional outcome was excellent and allowed the patient to resume pilot activity without any impairment (Figure 5).

### 3.5. Case 5

**Figure 6:**
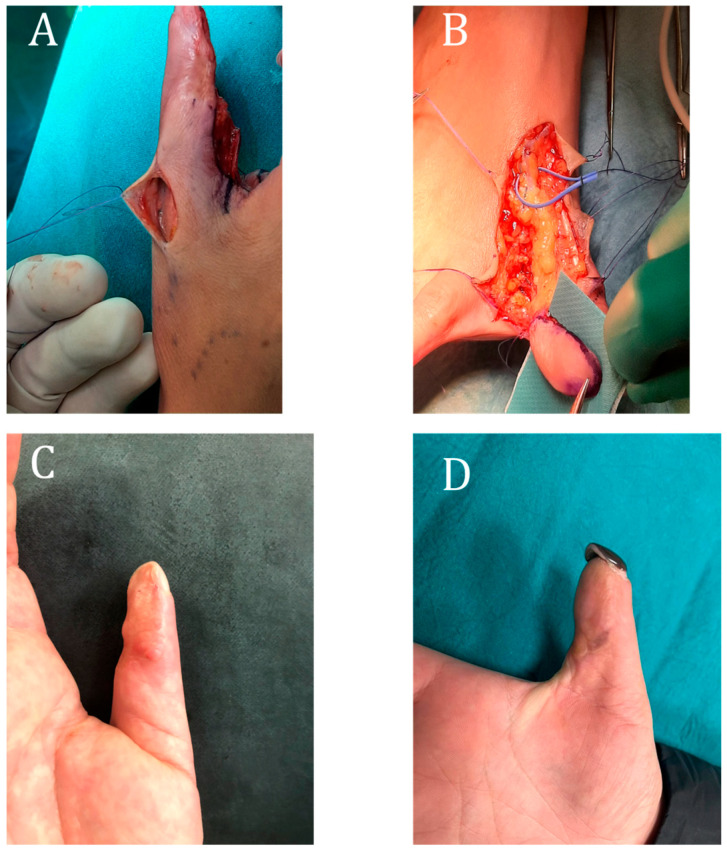
Case report 5: (**A**) preparation of the recipient site; (**B**) preparation of the harvest site; (**C**) atrophic outcomes of the thumb; (**D**) final result at follow-up.

A 38-year-old female manual worker patient presented with dystrophic findings at the level of the thumb pulp of the left hand (dominant). This was the result of a traffic accident that had occurred about 14 months earlier and was previously treated conservatively. The patient suffered from hyperalgesia, resulting in difficulty in performing work activities. A GPT flap was then performed; on the third day, reoperation was necessary due to thrombosis at the arterial anastomosis. No further intervention was subsequently necessary, and the patient completely resolved her pain symptoms with a complete work resumption (Figure 6).

## 4. Discussion

To the best of our knowledge, this is the first report in which a long-term follow-up was performed in patients who underwent toe pulp reconstruction surgery in scarred and atrophic outcomes via a neurocutaneous composite GTP flap. Only one other author has shown that chronic injuries can be treated with good results by using a neurocutaneous composite free flap from the foot. In this report, however, the second toe pulp was used as the donor site [14]. Nowadays, performing conservative treatment of digital pulp injuries with occlusive dressings or through the use of local flaps results in excellent healing in the majority of cases, making the digital pulp reconstruction procedure with more sophisticated methods necessary only in a small percentage of patients. Therefore, in view of the vast arsenal of digital pulp reconstruction techniques, foot-free flaps are a relatively rare indication and only in cases where the loss of substance is greater than 3 cm^2^ with flexor tendon and bone exposure [15,16]. In other cases, occlusive dressing with bone shortening or a dermal matrix makes for optimal results [17]. While this debate is open regarding acute injuries, nothing is reported in the literature about the treatment of retracting scars or pulp atrophy. For three patients the initial extent of the trauma was not clear, and therefore it was unclear which strategy was adopted for their treatment. It can be considered that an immediate local flap or appropriate wound management with early motion could have been beneficial for proper healing. The body’s attempt to repair the injury site resulted in a retracting scar that limited joint extension. When managing the loss of substance, it resulted in a bigger loss of tissue than would have been expected. So, after debridement and full extension of the PIP or IP joint, a loss of more than 3 cm^2^ resulted, requiring a free tissue transfer to reconstruct such a defect. In patient 3, the GTP flap solved two tasks. Firstly, the chronic ulcer could have been secondary to daily usage of the forearm skin for pinching, something that it is not made for. With the free flap, a specific skin designed for pressure bearing has been transferred into the fingertip position. Secondly a retracting scar limiting thumb abduction was released and substituted with the supple tissue of the flap tail. It is clear that, when dealing with a dystrophic fingertip, a flexion contracture of adjacent joints should be managed contemporarily, so that the physician has to figure out a bigger flap than one would expect, looking only at the skin. Patient 4 is midway between an acute and a chronic situation. In fact, a sub-amputation led to progressive necrosis of the pulp, with tendon and bone exposure without any chance of using a local flap due to gap extension. Such a situation could have resulted in a dystrophic scar, as for previous patients. The use of a GTP flap in a subacute setting prevented this. In one of our cases an acute arterial thrombosis occurred, and revision surgery was required to save the flap. This meant a longer hospital stay for the patient, but the outcome was not affected by this event. No venous graft was required for arterial anastomosis, showing that even in chronic cases digital arteries are available to efficiently revascularize the flap, even with a short pedicle, and it is not necessary to reach vessels in the palm or anatomic snuffbox for the thumb. Moreover, the complete survival rate of flaps in our series confirmed what is said in the literature [18,19,20,21,22]. Other than providing skin, a pulp flap is able to bring a sensate skin. The patients in our series presented values of the 2-PD test and the SM-MF in line with the results obtained by other authors who performed the acute reconstruction procedure. Yuan et al. [23], in their series of 24 cases, reported flap necrosis as a complication in four patients; the mean S-2PD test result was 5 mm with a mean follow-up of 20 months. In 2014, Gu et al. [24], in their series of pulp reconstructions using a free pulp flap of the big or second toe, reported a mean value of 4.8 mm at re-evaluation of patients at 20 months in the S-2PD test, and 4.12 in the SW-MF test. Subsequently, another Chinese author [25] published his series of 32 finger pulp reconstructions with a mean value of 6.17 mm in the S-2PD test and 3.675 in the SW-MF test at the mean follow-up of 22.8 months. Balan [26] reported a series of five acutely treated cases, in which he obtained a median value of 6.5 mm for the S-2PD and 4.31 for the SW-MF test at 9-month follow-up. Wang et al. [27], during a 34.8-month follow-up in a series of 34 patients, found a median value of 7.13 mm in the S-2PD test and 3.48–4.71 in the SW-MF test. Recently, an Italian group [28] in their series of 37 cases with a mean follow-up of one year obtained a mean S-2PD value of 9.41 mm; they concluded that, especially for more distal lesions, a technique without nerve suturing can be used. In our series, the recovery was comparable to previous reports, even when a complication occurred. Even in chronic cases, sensory recovery is expected to be good, providing an S3+ or S4 sensation. One should consider that several factors affecting sensation recovery such as age, follow-up period, and sensory re-education are known in the literature; in particular, a properly executed sensory rehabilitation program is essential for sensation restoration [29,30,31]. What nerve repair could be most useful for is the treatment and prevention of cold intolerance. In fact, it seems that nerve repair associated with vascular repair is able to reduce this symptom, sustaining nerve repair [32]. From this case series, it emerged that patients present only lesser symptoms related to cold intolerance, as presented in other studies [23,24,28]. Pulp reconstruction allowed patients to reach a normal hand function without any impediment to their work and daily activities. One of the main aspects of this reconstruction was the ability to solve a flexion contracture, greatly increasing the ROM of the affected finger and hand. Our results are limited due to the low number of cases in the study. But this fact could be explained by the relatively rare indication that this procedure has. In most cases, the appropriate debridement and treatment of fingertip injuries may prevent inappropriate healing. Furthermore, the dystrophic healing of the fingertip of an ulnar finger rarely required such a complex reconstruction, since it is better tolerated than those involving thumb and radial fingers. What can be deduced from our case series is that immediate and appropriate treatment of fingertip injuries should be planned to prevent complications and reduce the need for a free flap. Four of the patients that have been treated presented a dystrophic pulp as a consequence of a second intention healing in an injury that probably would have been successfully treated by an immediate local or free flap reconstruction. When dystrophic healing occurs, it is associated with a retracting scar that induces a flexion contracture in the adjacent joints. When debridement is performed and a normal extension of the IP is obtained, more soft tissue is to be provided to maintain a complete ROM. This excludes, in most of cases, at least in our series, the use of local flaps. Their dimensions could be limited compared to soft tissue loss and, not secondarily, most of them are non-innervated. Furthermore, the use of a distant flap has the advantage of not injuring the recipient digit that received multitissutal damage already. The main drawback consists of the risk of microsurgical failure, but this is low in specialized microsurgical centers. Moreover, local flaps should not be demonized and kept as plan B in the event of free flap failure.

## 5. Conclusions

When a dystrophic fingertip results from an inappropriate acute management, the GTP flap appears to be an excellent strategy to restore the specialized tissue of finger pulp and moreover, to bring supple tissue to correct the PIP flexion contracture or the small first web space contracture. It is mostly required for thumb and radial fingers’ reconstruction, especially in young patients or those who need high functional demands and/or present an extensive loss of substance that cannot be resolved with local flaps. Its use in acute cases can be limited to patients who present an extensive loss of soft tissue with bone and tendon exposure, otherwise appropriate soft tissue management with medication, early motion, and/or local flap could lead to good results without the need for microsurgical reconstruction.

## Figures and Tables

**Table 1:** Patient characteristics and surgical information. LoS, length of stay.

	Sex	Age (Years)	Location (Finger)	Dominant Hand	Time from Trauma (Months)	Previous Treatment	Flap Length (cm)	Flap Width (cm)	Flap Size (cm^2^)	Surgical Time (min)	LoS (Days)	Associated Procedures	Complications
Case 1	M	20	Thumb	Yes	12	Dressing	3	1.5	4.5	285	8	None	None
Case 2	F	17	Index	No	16	Dressing	3.5	2	7	320	6	Z-plasty	None
Case 3	M	38	Thumb	Yes	140	Flap	3	2	6	310	7	First web space release	None
Case 4	M	15	Index	Yes	2	Revascularization	3	2	6	290	6	Z-plasty (delayed)	None
Case 5	F	38	Thumb	Yes	14	Dressing	3.5	2	7	305	25	None	Arterial thrombosis
Mean		25.6			36.8		3.2	1.9	6.1	301	10.4		
Median		20			14		3	2	6	305	7		

**Table 2:** Clinical results. F-U, follow up; S2-PD, Static 2 points discrimination; D2-PD, Dinamic 2 points discrimination; SW-MP, Semmes–Weinstein monofilaments; q-DASH, Quick DASH; MH-O, Michigan Hand Outcomes questionnaire; FF-I, Foot Functional Index assessment; CISS, Cold Intolerance Symptom Severity; O-S, overall satisfaction.

		Operated Side				Non-Operated Side										
	F-U (Months)	S2-PD (mm)	D2-PD (mm)	SW-MP	Sensory Scale (S)	S2-PD (mm)	D2-PD (mm)	SW-MP	Sensory Scale (S)	VAS op Finger	VAS Donor Site	q-DASH	MH-O	FF-I	CISS	O-S
Case 1	66	8	6	3.61	3+	4	3	2.83	4	0	0	6.80%	16	0	16	10
Case 2	46	6	6	2.83	4	3	3	2.83	4	1	0	9.10%	19	0	18	8
Case 3	30	8	8	4.31	3+	5	4	3.61	4	0	0	9.10%	18	0	10	9
Case 4	26	6	6	3.61	4	4	3	2.83	4	0	0	6.80%	16	0	12	9
Case 5	12	6	6	4.31	3+	4	3	2.83	4	0	0	4.50%	15	0	12	10
Mean	36	6.8	6.4	3.734		4	3.2	2.986		0.2	0	7.26%	16.8	0	13.6	9.2
Median	30	6	6	3.61		4	3	2.83		0	0	6.80%	16	0	12	9

## Data Availability

The original contributions presented in this study are included in the article. Further inquiries can be directed to the corresponding author.
